# Public Health and Hygiene

**Published:** 1898-06

**Authors:** 


### PUBLIC HEALTH AND HYGIENE.

DR. THOMAS B. CARPENTER, of the Buffalo Health Department, has an exhaustive article on Garbage disposal in the May *Sanitarian*, and ends it by saying, in referring to systems: "It is safe to affirm that no perfectly satisfactory method for all times and all places has yet been devised."

---

THE *Sanitary Plumber* has discovered that the death-rate among plumbers from Bright's, phthisis, cancer and bronchitis is in excess of the average; from lead poisoning the mortality figure is placed at 19.

---

THE May *Sanitarian* has a report bearing out the statement of Dr. G. W. Goler, health officer of Rochester, that in formaldehyde disinfection, dryness of the air of the infected room is an essential factor.

